# ﻿*Sileneisabellae* (Caryophyllaceae), a new campion species from serpentine soils of Albania

**DOI:** 10.3897/phytokeys.227.100850

**Published:** 2023-06-05

**Authors:** Federico Selvi, Cristina Gonnelli, Elisabetta Bianchi

**Affiliations:** 1 Department of Agriculture, Food, Environment and Forestry, Laboratories of Botany, University of Florence, Piazzale delle Cascine 28, I-50144, Florence, Italy; 2 NBFC, National Biodiversity Future Center, I-90133, Palermo, Italy; 3 Department of Biology, University of Florence, via Micheli 1, I-50121, Italy

**Keywords:** Albanian flora, morphology, new species, serpentine endemism, *Silene*, taxonomy

## Abstract

The new species *Sileneisabellae* is described and illustrated from the Skënderbëut mountain range of central Albania. It grows on the ultramafic mountain slopes around Qafë Shtamë, in the understorey of open *Pinusnigra* forests and in the rocky grasslands above the forest belt, at 1000–1600 m a.s.l. *Sileneisabellae* is a serpentine endemic likely belonging to section Elisanthe (Fenzl ex Endl.) Ledeb. and shows affinities with the widespread European species *S.noctiflora* L. It is sharply distinct from the latter species in habit, stem and leaf pubescence, morphology, and biology of the flowers and length of the carpophore. Moreover, the ecology of the two taxa is also contrasting, being *S.noctiflora* a synanthropic-ruderal, mostly in lowlands. Weaker similarities were also observed with the south European subalpine taxa of the group of *S.vallesia* L. of section Auriculatae (Boiss.) Schischk., though these are not likely to reflect a real systematic affinity.

## ﻿Introduction

*Silene* L. is a large genus of family Caryophyllaceae Juss. including ca. 870 species of annual or perennial herbs, more rarely shrubs, that are mainly distributed in the Northern Hemisphere, south America and South Africa (Eggens et al. 2020; Jafari et al. 2020). Based on recent molecular phylogenetic evidence, the genus is subdivided into three subgenera and 44 sections (Jafari et al. 2020). About 200 native species are currently known from Europe (Mabberley 2017), which grow in temperate to Arctic regions and a wide range of environmental conditions, including forests and open habitats. A large part of the European species is found in the Mediterranean area and a high proportion of these are endemic (40% according to Greuter (1997)). In particular, the south-western countries of the Balkan Peninsula are major centres of species richness. According to Dimopoulos et al. (2018); (http://portal.cybertaxonomy.org/flora-greece/cdm_dataportal), a total of 140 taxa of *Silene* taxa (species and subspecies) is quoted for Greece, while the most recent checklist of the Albanian flora (Barina et al. 2018) lists as many as 52 native taxa (excluding erroneously reported and doubtful taxa). Several of these are stenoecious endemic to restricted areas, mountain ranges or particular geo-pedological conditions.

During field trips between 2006 and 2022, we had the opportunity to collect a large amount of plant material especially in the vast serpentine areas of internal Albania, which was the basis for floristic, systematic and taxonomic contributions to the Albanian flora (Cecchi et al. 2007; Cecchi and Selvi 2009; Coppi et al. 2014; Cecchi et al. 2018).

Amongst these collections, the specimens of a perennials *Silene* found on serpentine soils of the Skënderbëut Mountains (central Albania) were quite morphologically distinct. Concerning floral and seed characters, these plants approached *S.noctiflora* L. [Silenesect.Elisanthe (Fenzl ex Endl.) Ledeb.], an annual species of lowland and mostly ruderal habitats, which is widely distributed in central Europe, but unknown from Albania. Weak similarities were observed also with the subalpine taxa of the *S.vallesia* group [Silenesect.Auriculatae (Boiss.) Schischk.] of some mountain regions of southern Europe, including the Balkans (Ferrarini and Cecchi 2001). However, even stronger differences existed between the Albanian plant and these taxa. Based on the geographical isolation and ecological specialisation of the plant, we concluded that these specimens belong to a new species endemic to Albania, that is described here.

## ﻿Materials and methods

Identification of the specimens was first attempted using “Flora Europea” (Chater et al. 1993) as well as floras of Albania (Vangjeli 2015; Pils 2016) and Greece (Melzheimer 1986; Greuter 1997). We then went through whole lists of taxa of *Silene* reported in comprehensive checklists of the Albanian and Greek floras, especially Meyer (2011), Barina et al. (2018) and Flora of Greece web. Next, we examined material of *S.noctiflora* and the *S.vallesia* group kept in FI, FIAF and PAD, as well as scanned images of specimens from several virtual herbaria (BP, K, W, G). The herbarium acronyms follow Thiers (2023 [continuously updated]).

Specimens were morphologically examined with a Nikon stereomicroscope and a Dino-Lite & Dino-Eye digital microscope connected to DinoCapture 2.0 software vs. 1.5.38B, which was also used to take exact measurements of even small morphological structures, such as seeds and hairs.

Despite various attempts, seeds did not germinate and this prevented us from analysing chromosome features.

## ﻿Taxonomic treatment

### ﻿ Silene isabellae

Taxon classificationPlantaeCaryophyllalesCaryophyllaceae

Selvi & Bianchi sp. nov.

7326E116-DA81-573B-8B86-E79970E71B52

urn:lsid:ipni.org:names:77320611-1

Figs 1 , 2 , 3A–C

#### Type.

**Albania**, Krujë, verso Qafë Shtamë, pendice ripida del monte sopra la fonte lungo la strada prima del passo, sottobosco della foresta rada di *P.nigra*, suolo roccioso ultramafico (serpentino), 1100 m elev., 41°31.33'N, 19°53.27'E; *F. Selvi, E. Bianchi, I. Colzi & A. Coppi*, 12 Jul 2022 (holotype: FI068201; isotypes: K, G, TIR).

#### Diagnosis.

*Sileneisabellae* differs from *S.noctiflora* by the perennial habit with stoloniferous stems forming thick mats (instead of annual), the sparsely and shortly glandular-pubescent stem (vs. densely hairy), the basal leaves of the sterile stems present and widely obovate-spathulate (vs. absent), the cauline leaves linear-lanceolate (3–5 mm vs. ovate-lanceolate 25–35 mm wide), with shortly ciliate margins (vs. densely pubescent on both surfaces), the longer calyx teeth (ca. 8.5 vs. 7.0 mm), the corolla unscented and open during daytime (instead of scented and opening at evening), the petal lobes dentate (vs. entire), the fruiting calyx with prominent longitudinal ribs and the longer carpophore (6–7 vs. 2–3 mm).

#### Description.

Perennial herb forming thick mats, with branched stoloniferous stems thickened at the nodes. Flowering stems up to 40 cm, rigid, usually 1–4 branched in the upper half, rarely simple, shortly glandular-pubescent. Basal leaves mostly appressed to the ground, 30–80 × 8–18 mm, tapering into the petiole, widely obovate-spathulate, apiculate at tip; leaf blade thick, glabrous and finely papillose on both surfaces, shortly ciliate at the margins (hairs ca. 0.15 mm). Cauline leaves in pairs of 4–5, the uppermost narrowly lanceolate to almost linear, 30–50 × 3–5 mm, acuminate, the mid- and upper ones much shorter than the internodes, finely ciliate along margins with glandular and simple hairs on both surfaces. Inflorescence with 1–4 branches each bearing a single flower, rarely simple; branches finely glandular-pubescent, with two linear-lanceolate leaf-like bracts 6–8 mm long. Flowering calyx narrowly cylindrical, 25–30 mm, including teeth 8.2–8.6 mm, linear-lanceolate, surface glandular-pubescent, veins tinged with purplish, reticulate especially in the upper half; fruiting calyx obconical, truncate at the base and swollen at the middle, up to 13 mm wide, with 5–6 prominent longitudinal ribs. Corolla spreading 19–21 mm wide, petals 25–30 mm long (claw 15–20 mm long and slightly exserted), divided to ca. 1/3 in two lobes dentate at the outer margin, pale pink above, tinged with mauve red beneath; coronal scales exserted, truncate, nearly white; styles 3. Capsule smooth and glossy, ovoid-conical, 13–15 × 4–5 mm, dehiscing with 6 teeth curved outwards, carpophore 6–7 mm. Seeds ca. 1.1 × 0.83 mm, reniform, with surface finely papillose-tuberculate in concentric crests around the hilum, dark brown to blackish.

#### Etymology.

This species is dedicated to the first author’s wife, for her continuous support and advice during many botanical trips across the Mediterranean and the Middle East.

#### Phenology.

Flowering is from June to early July; fruiting is from end of June to end of July, depending on slope aspect and altitude.

#### Distribution and ecology.

*Sileneisabellae* is likely endemic to central Albania, precisely to the ultramafic sectors of the Skënderbëut mountain range running along the border between the districts of Krujë and Mat (Fig. 4). We could not observe it in other parts of this mountain range with calcareous soil. Additionally, we could not find it during our botanical trips across other ultramafic mountain areas of the country (amongst which were Tropojë, Pashtrik, Lurë, Bulqizë, Shebenik, Shpat, Moravë and Vallamarë), suggesting a restricted range and, possibly, the reason why this species has remained unknown until present. The plant is found in patches in the understorey of the open *Pinusnigra* forests and in the rocky grasslands on the mountain slopes around the pass Qafë Shtamë, between 1100 and 1600 m a.s.l., but possibly even higher (Fig. 2A, B). Slope aspect was prevalently south and south-west. This area is geologically characterised by vast outcrops of ultramafic rocks hosting a rich serpentine flora including remarkable Balkan endemics, such as *Forsythiaeuropaea* Degen & Bald., *Festucopsisserpentini* (C.E. Hubb.) Melderis and the Ni-hyperaccumulator *Odontarrhenasmolikana* (Bald.) Španiel, Al-Shehbaz, D.A.German & Marhold, subsp. glabra (Nyár. ex Markgr.) L.Cecchi & Selvi. We assume that *S.isabellae* is an obligate serpentine endemic.

#### Conservation status.

*Sileneisabellae* grows in a mountain area which has been subjected for a long time to grazing mainly by sheep and goats. This ancient land use form has implied the creation and conservation of large grassland areas at the expenses of the forest. Fire is also a recurrent disturbance especially to the pine forest on the mountain slopes. Over the area that we could visit (ca. 5 km^2^), the species was relatively frequent, though discontinuously distributed. Based on the present state of knowledge, its range and extent of occurrence seem quite restricted, suggesting categorising it as “nearly threatened” (NT), mainly according to IUCN criterion B (IUCN 2012). However, more field studies are needed to define the correct conservation category of this species.

#### Discussion.

**Figure 1.**
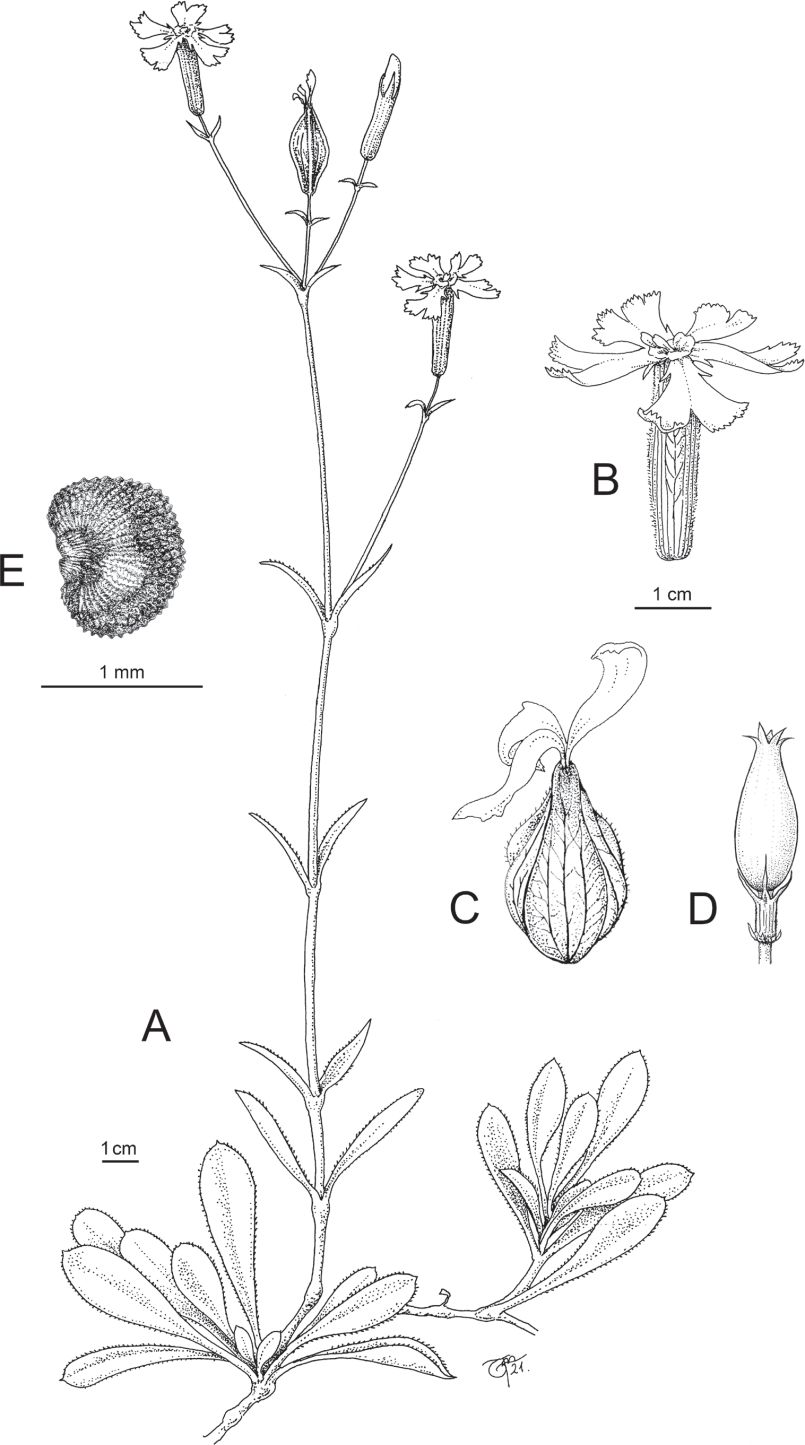
*Sileneisabellae***A** whole plant **B** flower with calyx and corolla **C** fruiting calyx **D** capsule and carpophore **E** seed. Original drawing by Laura Vivona.

**Figure 2.**
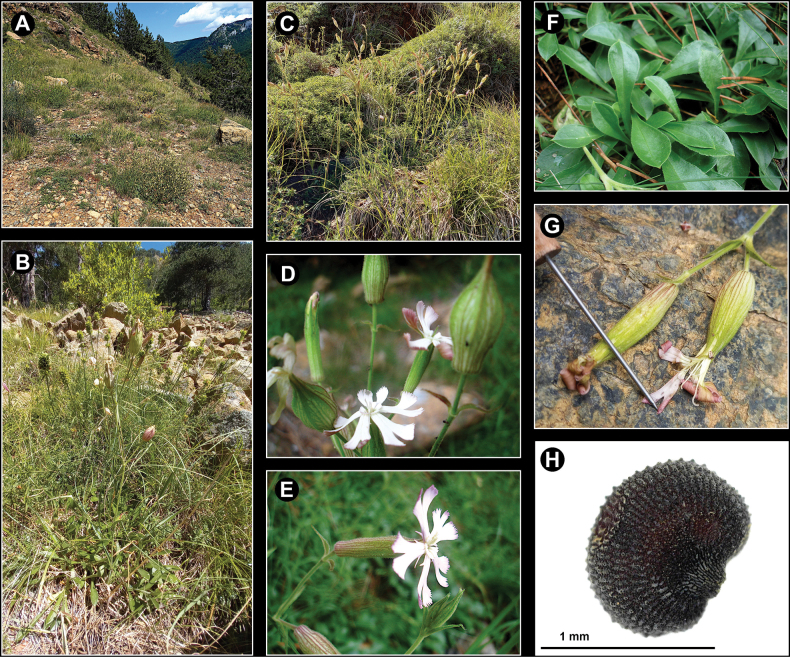
Field photos of *Sileneisabellae***A** habitat **B, C** whole plants in natural habitat **D** inflorescence with flowers and fruiting calyces **E** flower **F** basal leaves **G** flowers at late stage **H** seed.

*Sileneisabellae* shows morphological affinities with taxa of Silenesubg.Behenantha (Otth) Torr. & A. Gray sect. Elisanthe (Fenzl ex Endl.) Ledeb. and is assumed to belong here. This is a well-defined group supported by molecular evidence (Jafari et al. 2020) that includes annual, biennial and perennial species with large flowers, solitary or in dichasia, often dioecious, with bifid petal limb, prominent coronal scales, often five styles, capsule without basal septa dehiscing with 6 or 10 teeth and a very short carpophore (Chater et al. 1993). In Europe, the section includes widespread species, such as *S.latifolia* Poir., *S.dioica* (L.) Clairv. and *S.noctiflora* L. (Prentice 1978). The latter species, described by Linnaeus (1753) from Sweden and Germany (“Habitat in Suecia, Germania”; see Jonsell and Jarvis (1994)), is apparently the more closely related one to *S.isabellae*, with which it shares the thick stem, the few-flowered inflorescence, the texture and shape of the calyx, the usually hermaphrodite flowers with three styles and the reniform seeds with a nearly identical ornamentation pattern. On the other hand, *S.isabellae* differs from *S.noctiflora* in several striking characters (see Table 1). Most importantly, it is a perennial plant forming thick mats with sterile stoloniferous stems and basal leaves (Fig. 1A), while *S.noctiflora* is annual with usually a single stem and lacking basal leaves. The latter is also different in the dense and long pubescence of the lower leaves and stems which are lacking in *S.isabellae*, where the lower leaves are nearly glabrous and the lower stem is very shortly glandular-pubescent. The flowers of *S.isabellae* are open during daytime and unscented, while *S.noctiflora* is characterised by scented flowers with petals enrolled during daytime, opening in evening and night. Moreover, the petal lobes of the latter are entire or nearly so, while these are distinctly dentate in the Albanian plant (Figs 1B, 2D, E). The fruiting calyx is more distinctly truncate at the base and obconical, provided with prominent veins forming longitudinal ribs (Figs 1C, 2D). The capsule is borne by a longer carpophore (Fig. 1D). Additional evidence comes from the comparison of the chorological and ecological traits of the two taxa. In fact, *S.noctiflora* is widespread in Central and Eastern Europe (Jalas and Suominen 1988), including Serbia, Kosovo and Methojia, where it occurs south of Priština, on the eastern rim of the Kosovo Basin (Prodanović et al. 2016; Fig. 4). The plant is not known from Albania (according to Vangjeli (2015), Pils (2016); Barina et al. (2018)) and there is, thus, no range overlap with *S.isabellae*. Moreover, *S.noctiflora* is a weed usually growing in ruderal places, arable fields, cultivated ground and other synanthropic habitats, usually in lowland areas (Chater et al. 1993).

**Figure 3.**
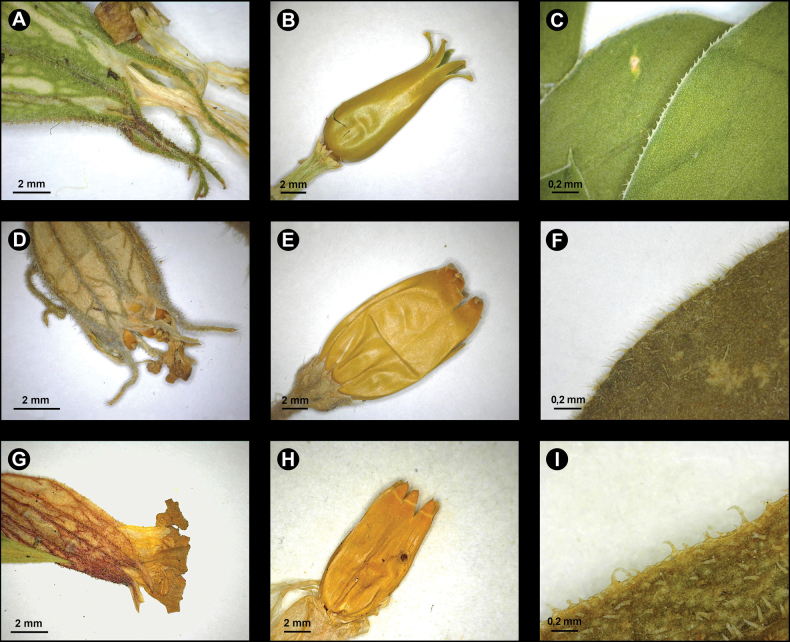
Calyx teeth, capsule, and margin of basal leaves of *Sileneisabellae* (respectively **A–C**), *S.noctiflora* (respectively **D–F**) and S.vallesiasubsp.graminea (respectively **G–I**).

**Figure 4.**
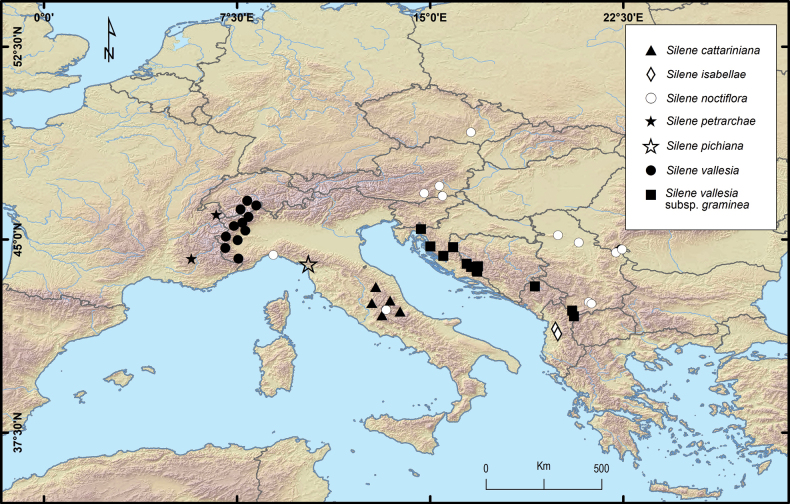
Distribution of *Sileneisabellae*, *S.noctiflora* (partial) and the taxa of the *S.vallesia* L. group.

**Table 1.** Main diagnostic morphological characters of *S.isabellae*, *S.noctiflora* and S.vallesiasubsp.graminea.

	*Sileneisabellae*	*Silenenoctiflora*	Silenevallesiasubsp.graminea
**Habit and life cycle**	perennial, mat-forming	annual, not mat-forming	perennial, mat-forming
**Flowering stems and branches**	sparsely and shortly glandular pubescent, 15–40 cm, usually branched, rarely simple, with 1–4 flowers	densely glandular pubescent, 12–50 cm, usually simple with a single flower, rarely branched with more flowers	sparsely pubescent, 5–15 cm, usually simple with a single flower
**Basal leaves of sterile stems**	widely obovate-spathulate, 8–18 mm wide, blade glabrous, but finely and densely ciliate along margins (Fig. 3C)	absent	linear 1.5–2 mm wide, blade with few simple hairs (Fig. 3I)
**Cauline leaves of flowering stems**	linear-lanceolate, 3–5 mm wide, shorter than internodes, shortly ciliate along the margins, blade with short glandular and simple hairs	ovate-lanceolate, up to 30 mm wide, blade and margins densely pubescent (Fig. 3F)	linear-lanceolate, 1.5–2 mm wide, subequal or longer than internodes, marginally ciliate at the base, with simple and few glandular hairs
**Flowers**	calyx 25–30 mm, teeth 8 mm, narrowly cylindrical in flower, truncate-obconical in fruit and prominently ribbed (Fig. 3A); corolla unscented, open during daytime; petals not enrolled during daytime pale pink above, tinged with mauve red beneath, lobes dentate	calyx 20–30 mm, teeth ca. 7 mm (Fig. 3D); attenuate at the base in fruit, not ribbed; corolla opening in evening with petals enrolled during daytime, pink above and yellowish beneath, lobes entire	calyx 12–16 mm, teeth ca. 1.8 mm, narrowly obconical and not ribbed in fruit (Fig. 3G); corolla unscented, open during daytime, petals usually white, lobes entire
**Fruits**	capsule conic-ovoid, 13–15 × 5 mm, surface smooth, glossy (Fig. 3B); carpophore 6–7 mm	capsule ovoid 13–15 × 8–10 mm, glossy (Fig. 3E); carpophore 2–3 mm	capsule conic-ovoid 12–13 × ca. 4 mm, glabrous, but finely rugose (Fig. 3H); carpophore 9 mm

*Sileneisabellae* shows a weak morphological affinity also with the taxa of the *S.vallesia* group [sect. Auriculatae (Boiss.) Schischk.]. This group includes taxa endemic to some mountain regions of southern Europe (Fig. 4), in France (*S.petrarchae* Ferrarini & Cecchi), the western Alps (S.vallesiaL.subsp.vallesia), the Apuan Alps in Italy (*S.pichiana* Ferrarini & Cecchi), the central Apennines (*S.cattariniana* Ferrarini & Cecchi) and the Balkan massifs [S.vallesiasubsp.graminifolia (Vis ex. Rchb.) Nyman, Ferrarini and Cecchi 2001]. Similarities are in the perennial habit with stoloniferous stems forming mats, the glandular-pubescent stems and leaves, the few-flowered inflorescence, the large flowers with narrowly cylindrical and reticulate-veined calyx, truncate at base, the corolla with bifid petals, the conical-ovoid capsule and the reniform seeds with a similar surface ornamentation. However, the only taxon of this group occurring in the Balkans and Albania, S.vallesiasubsp.graminea, is strongly differentiated from *S.isabellae* in the much smaller size of the plant, the shape of the leaves (narrowly linear-lanceolate), the usually single flower per stem and other features of the calyx teeth and capsule (Table 1, Fig. 3G). Moreover, this taxon is only found in the Alpine massifs of the north-east of the country in the district of Kükes and close to the border with Kosovo and Macedonia, such as Mt. Pashtrik, Mt. Gjallica and Mt. Koritnik, where it reaches the southern limit of its distribution. This taxon is, thus, likely to be allopatric and geographically isolated with respect to *S.isabellae*, which is found over 80 km to the southwest (Fig. 4). Based on Vangjeli (2015), S.vallesiasubsp.graminea is found in the alpine belt at 1700–2000 m a.s.l., thus, at higher elevation than *S.isabellae* and is typical of calcareous substrates especially limestone (Ferrarini and Cecchi 2001; Pils 2016).

*Sileneisabellae* is apparently found only on serpentine soils. Thanks to this specialisation, the plant can be categorised as a “metallophyte”, being able to cope with the typical chemical and physical anomalies of the serpentine soils and especially the poverty in nutrients, the high levels of Mg and in trace metals like Ni, Zn and Cr (Wójcik et al. 2017). We plan further studies to assess the concentration of these metals within the plant and, consequently, the strategies that it adopts to thrive on such hostile soils. Adaptation to serpentine soils is not uncommon in the genus *Silene*, being found in several other taxa of especially the Balkan flora. According to Stefanović et al. (2003), the Balkan countries host seven taxa of this genus that can be classified as obligate serpentine endemics, while others are found on serpentine as well as non-serpentine soils (the so-called “facultative serpentinophytes”). The distant phylogenetic position of these species suggests that ecological speciation driven by the “serpentine factor” has occurred through multiple and independent events, as in other plant groups of the Mediterranean and Balkan flora (Cecchi and Selvi 2009; Cecchi et al. 2011, 2013). Remarkably, some of the serpentine-tolerant species were also found at the same locality of *S.isabellae*, such as *S.paradoxa* L., *S.italica* L., *S.saxifraga* L., S.vulgarisL.subsp.prostrata (Gaud.) Schinz & Thell. and *S.damboldtiana* Greuter.

*Sileneisabellae* shows some variation in quantitative characters associated with site conditions. Plants from the coniferous forest at lower altitude (1000–1200 m a.s.l.) were characterised by taller flowering stems and larger leaves, in particular those of the sterile stems. In plants from open and rocky sites, we observed a general reduction in the size of the plants, also associated with altitude (above 1400 m a.s.l.), while qualitative characters remained constant.

#### Additional specimens examined.

***Sileneisabellae***: **Albania.** District of Krujë, Qafë Shtamë, rocce di serpentino nella foresta di *Pinusnigra*, ca. 800 prima del passo, 41°31.35'N, 19°53.32'E, 1100 m elev., 30 Jun 2018, *F. Selvi & I. Bettarini* (FI053966); District of Krujë, Qafe Shtamë, pascoli rupestri e margini di boscaglia rocciosa lungo la pista che sale dal passo verso Nord, suolo ultramafico (serpentino), 41.5251°N, 19.5251°E, 1150–1450 m elev., 12 July 2022, *F. Selvi, E. Bianchi, I. Colzi & A. Coppi* (FI068202).

***Silenenoctiflora*: Austria.** Kärnten. Lavanttal: auf dem Autobahn-Rastplatz in St. Andrä, Bezirk Wolfsberg, Gemeinde Sankt Andrä, Katastralgemeinde Kollegg, 46°47'08"N, 14°48'46"E, 450 m elev., 27 May 2008, *Helmut M.* s.n. (GJO 0041410); Österreich, Steiermark; Steirisches Randgebirge, Westliches Grazer Bergland; Bezirk Graz (Stadt); Gösting, Ruine Gösting, 47°06'13.44"N, 15°22'48.28"E, 541 m elev., 6 September 2010, *Leonhartsberger S*., (GJO 0062960); Windische Bühel, Kreuzberg bei Leutschach, Oberfahrenbach - Gamlitz - Karnerberg, Gasthof Eichbergho, 46.725°N, 15.45833°E, 07 November 1989, *Maurer W*. s.n., (GZU 000239138); **Belgium.** Moissons, Hackerinver, July 1863, *Thieleus Armand*; cultivè de cinq pieds sauvages trouvés en 1865 entre Haekendover (Brabant) et Overhespen (Liège), July 1866, (FI). **Czech Republic.** Bohemia, distr. Český Krumlov. VÚ Boletice: Nová Víska, okraj cesty přes louky 6 km JV od kostela v Křišťanově, 48.86556°N, 14.07°E, 14 July 2017, *Grulich V*. (BRNU 636522); Moravia centr., distr Brno: inter segtes ad pedem collis Zlobica dicti situ septentr-orientali ad oppido Kuřim, 280 m elev., 23 July 1974, *Dvořàk F*. (FI). **France.** Environs de Strasbourg, 1805, *Nestler C. G*. sn, (G00215384); Douai (Nord), abondant dans un jardin où il a peut-être ètè introduit accidentallement, 26 July 1882, *Maugin* (FI); Versailles, 17 August 1853, *Caruel L*. (FI). **Germany.** Thuring, 1822, *Wallroth K.F.W*. sn, (G00215386). **Italy.** Luoghi erbosi presso Cretone di Tornimparte (L’Aquila), 1200 m elev., 10 September 1971, *Pelliccione G*. (FI), Valle del Lagaccio, Liguria, June, *Baglietto* (FI); Cult. nell’Orto Botanico Sperimentale di Vallombrosa, 29 August 1894, *Solla n.* 1413 (FIAF); Giardino dei Semplici, 1830, *Ricasoli* (FI).

**Silenevallesiasubsp.graminea: Albania.** Velika Golia, 1700 m elev., 17 July 1904 (FI); Hais Patrik An Felsen in der Gipfelregion, 1800 m elev., 24 July 1877, *I. Dolfler* (W); Aufdem Berge Patrik bei Prizzen, 1900 m elev., 20–21 July 1936, *O.V.E. Behr* (FI); Prizren: Mt. Pastrik in graminosis alpinis, 21 July 1936, *Skrivanek* (BP591483); Distrikt Hasi, Pastrik, An Felsen in der Gipfelregion, 24 July 1918, *Dörfler & Ignaz* (BP455289, BP455288). **Bosnia and Herzegovina.** Gipfelregion der Golia: Sudskamn (?) der Velica Golja, 1700 m elev., 18 July, *J. Stdlmann & F. Faltis* (FI); KameKnica bai Livno, 1800 m elev., 27 July 1897, *E. Brandis* (PAD). **Croazia.** Ex rupibus Dinaricis ad …Dalmaticae, Herb Levier (FI); In saxosis apricis, Dinara, Ghnjat, Prologh, *Visiani* (PD); Mont Ghnjat (PAD); In dorso apricot et saxoso Mts. Dinara, July, 51K (Jos. Kargl) (PAD); Am Chemeschniza am Prolog, 18 July 1868, *Th Pichler* (PAD); Dalmatien M. Prolog, 18 July 1868, *Th. Pichler* (TSB); Croatia. Velebit in lapidosis herbosis m. Risioica supra Allau, alt. ca. 1400 m, 1908, *G. Lengyel* (PI042104); In montib. Dalmatia, s.d., *R. Visiani* (HAL0012278). **Montenegro.** In saxosis excelsioribus M. Durmitor, 870, *J. Panic* (PAD).

### ﻿Key to distinguish *Sileneisabellae* from *S.noctiflora*

**Table:** 

1	Perennial, with stoloniferous stems forming mats; basal leaves of the sterile stems widely obovate-spathulate, cauline leaves linear-lanceolate 3–5 mm wide, with sparse and short hairs; fruiting calyx longitudinally ribbed in fruit with teeth ca. 8.5 mm long; corolla unscented, open during daytime, petals not enrolled during daytime, with lobes dentate; carpophore 6–7 mm	***S.isabellae***
–	Annual without sterile stoloniferous stems; basal leaves absent, cauline leaves ovate-lanceolate, up to 30 mm wide, densely pubescent; fruiting calyx not ribbed in fruit, with teeth ca. 7 mm; corolla scented, opening in evening with petals enrolled during daytime, petal lobes not dentate, carpophore 2–3 mm	***S.noctiflora***

## Supplementary Material

XML Treatment for Silene isabellae

## ﻿Additional information

### Author ORCIDs

Federico Selvi https://orcid.org/0000-0002-3820-125X

Cristina Gonnelli https://orcid.org/0000-0001-5201-586X

Elisabetta Bianchi https://orcid.org/0000-0003-1197-4081

### Data availability

All of the data that support the findings of this study are available in the main text or Supplementary Information.
